# What Shall We Eat?

**Published:** 1894-09

**Authors:** 


### What Shall we Eat?

Every once in a while there is a recrudescence of vegetarianism and also many reappearances of special recommendations as to diet that are so extreme, not to say "cranky," as to suggest that the persons making them must have spoken out of the sufferings of stomachs so far from normal as rightly to be called "cranky."

The traveller from a distant planet, returning from his home and describing our habits, would say: "They prey upon other animals." It has a repulsive sound; but, of course, it is just what meat-eaters do, in spite of all the refinement and transformations that, as Emerson says, "intervene between the slaughter-house and our plates," where the slice of steak or roast reposes so tranquilly as to give no hint that it was once alive and as full of sensitive nerves as is the mouse on which the cat so ruthlessly pounces.

But let even the hypersensitive founders of the Society for the Prevention of Cruelty to Animals reflect a moment, and ask themselves if the deliberate killing of an animal, by intelligent and humane methods, is not a more merciful fate than the slow perishing by disease or old age, which certainly would occur in a state of unassisted nature.

At the two large abattoirs of Lyons, France, the guards protect the animals to be slaughtered from seeing anything connected with the slaughtering of other animals, as terror is found to have an injurious effect upon the secretions and flesh of dumb creatures. Zurich, in Switzerland, twenty years ago, invented a merciful way of killing cattle; a leathern strap completely covering the eyes is slipped over the animal's horns before he is taken to the slaughter-house. In the centre of this strap is a perforated iron block, the hole being directly over the centre of the forehead. Standing in this hole is a short, sharp, hollow, steel spike, which is driven into the brain by a single blow of a heavy hammer, producing instant unconsciousness. Immediately a sharp iron rod is made to penetrate that point in the base of the brain which corresponds to the "vital knot" of the human system, and absolute death is the result. The large blood vessels are then severed. As civilization advances, we see every-where more humane laws being enacted for what must be considered the *indispensable killing of animals*, if man is to continue on his upward career in knowledge, wisdom and happiness; for the brain-worker, far more certainly than the muscle-worker, must have flesh food to work upon.

The vegetarian can point to a considerable array of facts that sustain this theory. Many tribes of North American Indians have attained a very high physical development upon a vegetable diet; many Scotch farmers live principally upon oat-meal; meat is a comparative rarity among European laborers. Many Englishmen formerly lived on bread, cheese and beer (though cheese can hardly be called a vegetable); the Roman legionary carried a weight of sixty pounds and performed astounding feats of strength and endurance without eating flesh, and some people have substituted eggs and milk for meat, under the delusion that they are less atrocious than flesh-eaters; but the opinion of the cow whose calf they have robbed of its natural food should be asked before final judgment.

Dr. C. N. Folsom, then Secretary of the Massachusetts Board of Health, wrote twenty years ago:

"The Brahmins of India eat nothing which contains the germ of animal life, although they can hardly be said to have attained a vigorous physical or mental culture."

Mr. Edison puts the truth here hinted at more tersely in "Those who eat rice think rice," while Dr. Folsom still further says:

"Although physiologists are not agreed that *animal* food is absolutely essential to a high degree of civilization, there are certainly many facts which seem to indicate that it is resolvable into a greater amount of force than other nitrogenous foods."

That the kind and quality of food gets into a man's thought and disposition is not to be denied. The physicians at Sebastopol, after the Crimean war, testified that when a Russian soldier was intoxicated he became disgustingly maudlin, affectionate and silly, while the Briton in that state became at once "full of fight," and they accounted for it by the fact that the first lived on black bread and the second had meat. Dogs that are quiet and subdued on vegetable food grow fierce on meat, and there are persons of fine nervous organizations who find in meat a stimulus similar to that of wine.

The great advance in analytical chemistry in these last two decades has done much to reveal wherein the "potential energy" of foodstuffs lies, and the "training" of our athletes shows that we are beginning to prescribe food with as much precision as we can use the most familiar drugs—say as quinine and opium.

When James Russell Lowell went out to "rough it" among the Maine lumbermen, he was astonished to find that the essential article of their diet was fat pork, against which he at that time held a genteel prejudice; a few days of heavy tramping brought him to a complete appreciation of its merits as a heat and strength producer. These lumbermen labor intensely in the cold and snows of winter and in the icy water in spring, and beans and fat pork are the staple of their diet, and the modern chemist comes along with his physiological yardstick and tells us the exact reason why men performing the several tasks prefer pork to the choicest cuts of beef.

"The energy from the sun is stored in the protein and fats and carbohydrates of food, and the physiologists of to-day are telling us how it is transmuted into the heat that warms our bodies, and into strength for our work and thought," (Prof. W. O. Atwater).

Professor Frankland determined the heats of combustion of many substances, and reckoning them in units called "calorics." Among forty-five substances tested, he found that very fat pork contained an amount represented by 3,452, while fat beef contained 2,750, lean beef 807, and turnips only 139, while beans came up to 1,519, and peas to 1,476. Many of us do not require so much "energy" in our food, but we do need material which will restore the worn-out and ever wearing-out tissues of the body, and for this we must have foods that contain much protein, and these are beef, some kinds of fish, and eggs. All food substances have more or less of water, but the driest of all is fat pork. For a most interesting study of the whole subject, where the relative value of different substances for nutrition is shown by an ingenious diagram at a glance, the reader is referred to a series of articles by Prof. W. O. Atwater, in the *Century Magazine*, beginning in May, 1887.—*Independent.*

